# Environmental Shotgun Sequencing: Its Potential and Challenges for Studying the Hidden World of Microbes

**DOI:** 10.1371/journal.pbio.0050082

**Published:** 2007-03-13

**Authors:** Jonathan A Eisen

## Abstract

Environmental shotgun sequencing promises to reveal novel and fundamental insights into the hidden world of microbes, but the complexity of analysis required to realize this potential poses unique interdisciplinary challenges.

**Figure:**
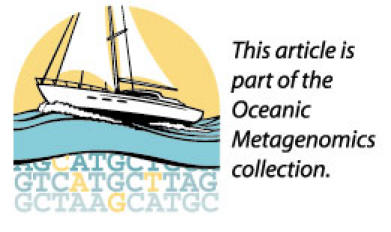


Since their discovery in the 1670s by Anton van Leeuwenhoek, an incredible amount has been learned about microorganisms and their importance to human health, agriculture, industry, ecosystem functioning, global biogeochemical cycles, and the origin and evolution of life. Nevertheless, it is what is not known that is most astonishing. For example, though there are certainly at least 10 million species of bacteria, only a few thousand have been formally described [1]. This contrasts with the more than 350,000 described species of beetles [2]. This is one of many examples indicative of the general difficulties encountered in studying organisms that we cannot readily see or collect in large samples for future analyses. It is thus not surprising that most major advances in microbiology can be traced to methodological advances rather than scientific discoveries per se.

Examples of these key revolutionary methods (Table 1) include the use of microscopes to view microbial cells, the growth of single types of organisms in the lab in isolation from other types (culturing), the comparison of ribosomal RNA (rRNA) genes to construct the first tree of life that included microbes [3], the use of the polymerase chain reaction (PCR) [4] to clone rRNA genes from organisms without culturing them [5–7], and the use of high-throughput “shotgun” methods to sequence the genomes of cultured species [8]. We are now in the midst of another such revolution—this one driven by the use of genome sequencing methods to study microbes directly in their natural habitats, an approach known as metagenomics, environmental genomics, or community genomics [9].

**Table 1:**
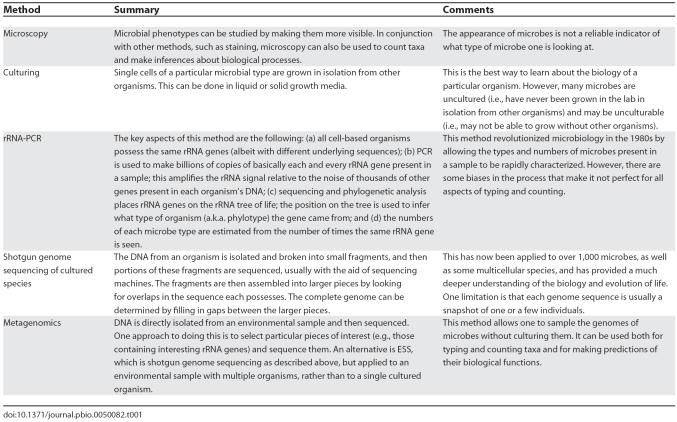
Some Major Methods for Studying Individual Microbes Found in the Environment

In this essay I focus on one particularly promising area of metagenomics—the use of shotgun genome methods to sequence random fragments of DNA from microbes in an environmental sample. The randomness and breadth of this environmental shotgun sequencing (ESS)—first used only a few years ago [10,11] and now being used to assay every microbial system imaginable from the human gut [12] to waste water sludge [13]—has the potential to reveal novel and fundamental insights into the hidden world of microbes and their impact on our world. However, the complexity of analysis required to realize this potential poses unique interdisciplinary challenges, challenges that make the approach both fascinating and frustrating in equal measure.

## Who Is Out There? Typing and Counting Microbes in the Environment

One of the most important and conceptually straightforward steps in studying any ecosystem involves cataloging the types of organisms and the numbers of each type. For a long time, such typing and counting was an almost insurmountable problem in microbiology. This is largely because physical appearance does not provide a valid taxonomic picture in microbes. Appearance evolves so rapidly that two closely related taxa may look wildly different and two distantly related taxa may look the same. This vexing problem was partially overcome in the 1980s through the use of rRNA-PCR (Table 1). This method allows microorganisms in a sample to be phylogenetically typed and counted based on the sequence of their rRNA genes, genes that are present in all cell-based organisms. In essence, a database of rRNA sequences [14,15] from known organisms functions like a bird field guide, and finding a rRNA-PCR product is akin to seeing a bird through binoculars. Rather than counting species, this approach focuses on “phylotypes,” which are defined as organisms whose rRNA sequences are very similar to each other (a cutoff of >97% or >99% identical is frequently used). The ability to use phylotyping to determine who was out there in any microbial sample has revolutionized environmental microbiology [16], led to many discoveries [e.g., 17], and convinced many people (myself included) to become microbiologists.

The selective targeting of a single gene makes rRNA-PCR an efficient method for deep community sampling [18]. However, this efficiency comes with limitations, most of which are complemented or circumvented by the randomness and breadth of ESS. For example, examination of the random samples of rRNA sequences obtained through ESS has already led to the discovery of new taxa—taxa that were completely missed by PCR because of its inability to sample all taxa equally well (e.g., [19]). In addition, ESS provides the first robust sampling of genes other than rRNA, and many of these genes can be more useful for some aspects of typing and counting. Some universal protein coding genes are better than rRNA both for distinguishing closely related strains (because of third position variation in codons) and for estimating numbers of individuals (because they vary less in copy number between species than do rRNA genes) [10]. Perhaps most significantly, ESS is providing groundbreaking insights into the diversity of viruses [20,21], which lack rRNA genes and thus were left out of the previous revolution.

Certainly, many challenges remain before we can fully realize the potential of ESS for the typing and counting of species, including making automated yet accurate phylogenetic trees of every gene, determining which genes are most useful for which taxa, combining data from different genes even when we do not know if they come from the same organisms, building up databases of genes other than rRNA, and making up for the lack of depth of sampling. If these challenges are met, ESS has the potential to rewrite much of what we thought we knew about the phylogenetic diversity of microbial life.

## What Are They Doing? Top Down and Bottom Up Approaches to Understanding Functions in Communities

A community is, of course, more than a list of types of organisms. One approach to understanding the properties and functioning of a microbial community is to start with studies of the different types of organisms and build up from these individuals to the community. Ideally, to do this one would culture each of the phylotypes and study its properties in the lab. Unfortunately, many, if not most, key microbes have not yet been cultured [22]. Thus, for many years, the only alternative was to make predictions about the biology of particular phylotypes based on what was known about related organisms. Unfortunately, this too does not work well for microbes since very closely related organisms frequently have major biological differences. For example, Escherichia coli K12 and E. coli O157:H7 are strains of the same species (and considered to be the same phylotype), with genomes containing only about 4,000 genes, yet each possesses hundreds of functionally important genes not seen in the other strain [23]. Such differences are routine in microbes, and thus one cannot make any useful inferences about what particular phylotypes are doing (e.g., type of metabolism, growth properties, role in nutrient cycling, or pathogenicity) based on the activities of their relatives.

These difficulties—the inability to culture most microbes and the functional disparities between close relatives—led to one of the first kinds of metagenomic analyses, wherein predictions of function were made from analysis of the sequence of large DNA fragments from representatives of known phylotypes. This approach has provided some stunning insights, such as the discovery of a novel form of phototrophy in the oceans [24]. However, this large insert approach has the same limitation as predicting properties from characterized relatives—a single cell cannot possibly represent the biological functions of all members of a phylotype.

ESS provides an alternative, more global way of assessing biological functions in microbial communities. As when using the large insert approach, functions can be predicted from sequences. However, in this case the predicted functions represent a random sampling of those encoded in the genomes of all the organisms present. This approach has unquestionably been wildly successful in terms of gene discovery. For example, analysis of ESS data has revealed novel forms of every type of gene family examined, as well as a great number of completely novel families (e.g., [25]). However, there is a major caveat when using ESS data to make community-level inferences. Ecosystems are more than just a bag of genes—they are made up of compartments (e.g., cells, chromosomes, and species), and these compartments matter. The key challenge in analyzing ESS data is to sort the DNA fragments (which are usually less than 1,000 base pairs long relative to genome sizes of millions or billions of bases) into bins that correspond to compartments in the system being studied.

A recent study by myself and colleagues illustrates the importance of compartments when interpreting ESS data. When we analyzed ESS data from symbionts living inside the gut of the glassy-winged sharpshooter (an insect that has a nutrient-limited diet), we were able to bin the data to two distinct symbionts [26]. We then could infer from those data that one of the symbionts synthesizes amino acids for the host while the other synthesizes the needed vitamins and cofactors. Modeling and understanding of this ecosystem are greatly enhanced by the demonstration of this complementary division of labor, in comparison to simply knowing that amino acids, vitamins, and cofactors are made by “symbionts.”

How does one go about binning ESS data? A variety of approaches have been developed, some of which are described in Table 2. In considering the different binning methods and their limitations, the first question one needs to ask is, what are we trying to bin? Is it fragments from the same chromosome from a single cell, which would be useful for studying chromosome structure? If so, then perhaps genome assembly methods are the best. What if instead, as in the sharpshooter example, we are trying to have each bin include every fragment that came from a particular species, knowledge which may be useful for predicting community metabolic potential? If the level of genetic polymorphism among individual cells from the same species is high, then genome assembly methods may not work well (the polymorphisms will break up assemblies). A better approach might be to look for species-specific “word” frequencies in the DNA, such as ones created by patterns in codon usage. The challenge is, how do we tune the methods to find the right target level of resolution? If we are too stringent, most bins will include only a few fragments. But if we are too relaxed, we will create artificial constructs that may prove biologically misleading, such as grouping together sequences from different species. To make matters more complex, most likely the stringency needed will vary for different taxa present in the sample.

**Table 2:**
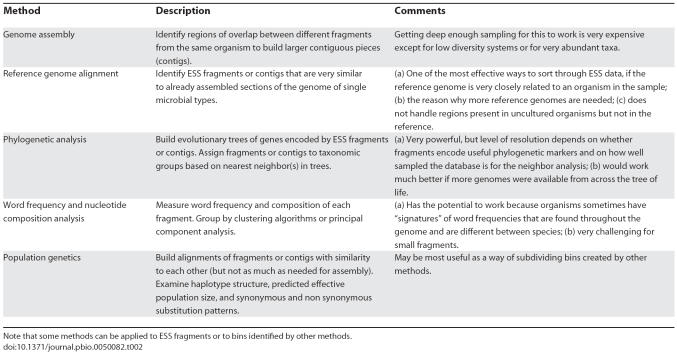
Methods of Binning

Another critical issue is the diversity of the system under study. Generally, binning works better when there are few different phylotypes present, all of which are distantly related and form discrete populations. This is why binning works well for the sharpshooter system and other relatively isolated, low diversity environments. Binning increases in difficulty exponentially as the number of species increases: the populations and species start to merge together, and the populations get more and more polymorphic and variable in relative abundance (such as in the paper about the Global Ocean Sampling expedition in this issue [27]). Further complicating binning is the phenomenon of lateral gene transfer, where genes are exchanged between distantly related lineages at rates that are high enough that random sampling of a genome will frequently include genes with multiple histories.

Despite these challenges, I believe we can develop effective binning methods for complex communities. First, we can combine different approaches together, such as using one method to sort in a relaxed manner and then using another to subdivide the bins provided by the first method. Second, we can incorporate new approaches such as population genetics into the analysis [28]. In addition, the lessons learned here can be applied to other aspects of metagenomics (e.g., the counting and typing discussed above) and provide insights into the nature of microbial genomes and the structure of microbial populations and communities.

## Comparative Metagenomics

So far, I have discussed issues relating mostly to intrasample analysis of ESS data. However, the area with perhaps the most promise involves the comparative analysis of different samples. This work parallels the comparative analysis of genomes of cultured species. Initial studies of that type compared distantly related taxa with enormous biological differences. What has been learned from these studies pertains mostly to core housekeeping functions, such as translation and DNA metabolism, and to other very ancient processes [29,30]. It was not until comparisons were made between closely related organisms that we began to understand events that occurred on shorter time scales, such as selection, gene transfer, and mutation processes [31]. Similarly, the initial comparisons of ESS data involved comparisons of wildly different environments [32], yielding insights into the general structure of communities. But as more comparisons are made between similar communities [33,34], such as those sampled during vertical and horizontal ocean transects [27,35–37], we will begin to learn about shorter time scale processes such as migration, speciation, extinction, responses to disturbance, and succession. It is from a combination of both approaches—comparing both similar and very divergent communities—that we will be able to understand the fundamental rules of microbial ecology and how they relate to ecological principles seen in macro-organisms.

## Conclusions

In promoting some of the exciting opportunities with ESS, I do not want to give the impression that it is flawless. It is helpful in this respect to compare ESS to the Internet. As with the Internet, ESS is a global portal for looking at what occurs in a previously hidden world. Making sense of it requires one to sort through massive, random, fragmented collections of bits of information. Such searches need to be done with caution because any time you analyze such a large amount of data patterns can be found. In addition, as with the Internet, there is certainly some hype associated with ESS that gives relatively trivial findings more attention than they deserve. Overall, though, I believe the hype is deserved. As long as we treat ESS as a strong complement to existing methods, and we build the tools and databases necessary for people to use the information, it will live up to its revolutionary potential.

## Abbreviations

ESS: environmental shotgun sequencing
PCR: polymerase chain reaction
rRNA: ribosomal RNA
